# Is it pathological to believe conspiracy theories?

**DOI:** 10.1177/13634615231187243

**Published:** 2023-08-01

**Authors:** Lisa Bortolotti

**Affiliations:** Philosophy Department and Institute for Mental Health, 1724 University of Birmingham, Edgbaston, UK

**Keywords:** cognitive dysfunction, conspiracy theories, implausibility, pathology, unshakeability

## Abstract

According to a naturalist conception of what counts as a disorder, conspiracy beliefs are pathological beliefs if they are the outcome of a cognitive dysfunction. In this article, I take issue with the view that it is pathological to believe a conspiracy theory. After reviewing several approaches to the aetiology of conspiracy beliefs, I find that no approach compels us to view conspiracy beliefs as the outcome of a dysfunction: a speaker's conspiracy beliefs can appear as implausible and unshakeable to an interpreter, but in a naturalist framework it is not pathological for the speaker to adopt and maintain such beliefs.

## Pathology as dysfunction

In recent years, beliefs in conspiracy theories (hereafter, *conspiracy beliefs*) have been compared to clinical delusions, in terms of their surface features, aetiology, and downstream effects. There are some similarities between the two in terms of surface features: both clinical delusions and conspiracy beliefs are strenuously resistant to counterevidence and are rarely abandoned under the pressure of external challenges. There are also some marked differences in downstream effects: clinical delusions are typically associated with distress and social isolation whereas conspiracy beliefs do not typically cause distress and can even strengthen bonds between members of well-defined social groups (see, for instance, Bortolotti et al., 2021; but see Jolley & Douglas, 2014, for an overview of the social costs of conspiracy beliefs).

What about aetiology? When a belief is described as *delusional*, the implication often is that the belief is pathological: the person reporting the belief is not in their right mind or their brains are not working properly. When authors compare the aetiologies of conspiracy beliefs and delusions, and find some overlap (as in Miller, 2020), the intention is often to pathologise the phenomenon of believing a conspiracy theory. By suggesting that conspiracy beliefs are like delusional beliefs in the ways in which they are formed and maintained, the implication is that a cognitive dysfunction is responsible for both, and conspiracy beliefs, just like delusional beliefs, are pathological. This argumentative strategy, however, is not compelling. The pathological nature of delusional beliefs is itself something that deserves to be explored further as even researchers who agree that delusions are malfunctioning beliefs disagree on what the dysfunction is (see, for instance, Miyazono, 2015; Sakakibara, 2016; and Petrolini, 2017). Moreover, there have been recent challenges to the view that delusions are pathological beliefs (see, for instance, Bortolotti, 2022).

Pathologisation has consequences and should not be embraced lightly: if the belief is considered to be the outcome of a dysfunction, then the person reporting it may not be taken seriously. Rather than the belief being challenged and argued against, it may be merely treated as the symptom of a disorder—as something to get rid of by attempting to restore functionality in the cognitive mechanisms responsible for the adoption and maintenance of beliefs. Another consequence is exclusion. People who report conspiracy beliefs may be prevented from participating in public debates and their views may be discredited as the product of “a sick mind”. But why do we think that a belief caused by a cognitively dysfunctional process is pathological?

An influential conception of disorder in the philosophy of medicine, *naturalism about disorder*, says that a pathology *is* a dysfunction (e.g., Boorse, 1977) and that pathologies can be identified by using the resources of science, that is, in a largely value-free way. The standard example in the literature is the heart failure. When people have coronary heart disease, fatty plaques narrow or block the coronary artery used by the supply of oxygen-rich blood to reach the heart muscle. When there is no sufficient flow of oxygen-rich blood, the heart muscle cannot contract properly, and people may experience a heart attack. According to naturalism, what makes coronary heart disease a pathology is that some important biological functions are not being carried out as they should: the coronary artery should supply sufficient oxygen to the heart muscle but it does not; the heart muscle should contract enough to supply oxygen to the rest of the body but it does not. By analogy, beliefs would be pathological when they are produced and sustained by dysfunctional cognitive processes.

But it is surprisingly difficult to show that a belief is caused by a cognitive dysfunction: even when we look closely at how clinical delusions are likely to be formed and maintained, it is far from obvious that they are the output of a dysfunctional process in a way that justifies their being characterised as pathological beliefs. Instead, the psychological evidence suggests that clinical delusions and conspiracy beliefs alike are outputs of biased cognition.

How does a *cognitive bias* differ from a *cognitive dysfunction*? There are numerous and conflicting definitions of what a cognitive bias is, but in several sophisticated accounts the presence of a bias does not imply that the cognitive output is mistaken or irrational and certainly does not imply that the cognitive process is failing to fulfil its function. For instance, in their contribution to the *Encyclopedia of Behavioral Neuroscience*, cognitive biases are defined as “systematic cognitive dispositions or inclinations in human thinking and reasoning that often do not comply with the tenets of logic, probability reasoning, and plausibility” (Korteling & Toet, 2022, p. 610). The fact that the dispositions do not conform to formal and abstract norms of reasoning does not mean that they signal a dysfunction; rather, as Korteling and Toet argue, biases are often characterised as *design features* in evolutionary frameworks.

In this article, I hope to show that there is no clear role for dysfunctional processes in the formation and maintenance of conspiracy beliefs. Conspiracy beliefs are the imperfect response to psychological and epistemic needs that people experience when facing a significant event that calls for an explanation. Conspiracy beliefs help people make sense of events that can be perceived as threatening and distressing but also enable people to see themselves as sensible and well-informed agents rather than passive bystanders when a crisis occurs. So, the adoption of conspiracy beliefs is in some sense a reaffirmation of agency and a contribution to an agent's personal and social identities.

However, as a response, conspiracy beliefs are imperfect, because they offer an epistemically inadequate explanation for the gap people aim to fill. The explanations people adopt when they believe a conspiracy theory may be easy to understand, engaging, and coherent with some aspects of their accepted worldview, but they often conflict with information provided by credible and authoritative sources. By leaving agents with an incomplete and often inaccurate map of the world, conspiracy beliefs may undermine agency and prevent people from pursuing and fulfilling some of the goals they set up for themselves. In this complex picture, conspiracy beliefs are not by default pathological beliefs, and may at the same time support and hinder agency (Bortolotti, 2023).

## How we adopt and maintain beliefs

How plausible it is that conspiracy beliefs are due to a cognitive dysfunction depends on our preferred theory of belief formation and maintenance. Competing theories reach different conclusions about whether a dysfunction is involved and, if so, where exactly the dysfunction lies. I am going to briefly consider here psychodynamic theories, predictive coding theories, and one- and two-factor theories of the formation and maintenance of conspiracy beliefs. This is not an exhaustive list but includes some of the most influential approaches to delusional and conspiracy beliefs.

Not all the approaches briefly reviewed here operate at the same explanatory level and should necessarily be seen as incompatible with one another. Some theories offer a *psychological* account of how we adopt beliefs, asking how discerning subjects explain puzzling experience and weigh up evidence. Other theories offer *neurobiological* accounts, referring to subpersonal processes by which information is gained and models of the world are updated. Such processes are not something agents are conscious of and deliberate about.

The approaches discussed here may be compatible (fully or in part) with one another and do not need to be seen as competitors. For instance, they may be used in combination in so far as they point to plausible factors that make a distinct contribution to the adoption of conspiracy beliefs. A woman's belief that immunisation may give her child autism can be arrived at and sustained for different reasons. She may have a history of bad experiences with medical professionals in her life that causes her to mistrust the advice provided by health practitioners and reject most invasive medical interventions. She may also have a personality that renders her more suspicious of authorities, and more vulnerable to conspiratorial thinking. In a recent study (Hornsey, 2021), vaccine hesitancy was found to correlate strongly with both *conspiracy mentality* (the tendency to accept a general worldview according to which elite groups and powerful individuals deceive the public by plotting secretly) and *identity expression* (the tendency to accept a view that coheres well with other views a person is already committed to). There is no reason to think that heterogeneous factors cannot be combined.

Biases affecting information processing can also be part of the picture. In a recent attempt to map the cognitive biases that might affect vaccine hesitancy and anti-vaccination beliefs (Azarpanah et al., 2021), three types of biases were examined: biases triggered by information about vaccines (such as the availability bias); biases triggered by the decision whether to vaccinate (such as the optimism bias); and biases triggered by prior beliefs (such as the confirmation bias). Due to the *availability bias*, a recent report in the media of a child dying as a result of a reaction to immunisation is more vivid, easier to remember, and more influential in the woman's decision-making process than the copious data about the safety of immunisation programmes. Due to the *optimism bias*, the woman might come believe that the disease her child should be immunised against is not going to pose a threat to her child even if it poses threats to other people. This leads her to believe that it is not necessary for her child to be immunised against that disease. Due to the *confirmation bias*, if the woman already tends to mistrust doctors and is concerned about the risks of medical interventions, she is more likely to dismiss arguments about the utility and overall safety of vaccines.

## Conspiracy belief as a defence from an undesirable reality

Psychodynamic and motivational accounts are no longer popular, but they used to be the standard explanation of delusional beliefs and they have recently become influential again in some specific contexts (see, for instance, the work by McKay et al., 2005; and Fotopoulou, 2008). The fundamental idea is that unusual beliefs emerge when the actual reality is overwhelming, and the person cannot cope with it any longer. The belief consists in an alteration of reality that may be in some respects less difficult to cope with, or more desirable, than the actual reality (de Pauw, 1994). In its general formulation, the psychodynamic approach is difficult to test empirically but it has been applied to delusional beliefs that at least temporarily support the person's self-esteem and sense of self-worth.

On a psychodynamic story, some of the mechanisms motivating an unusual belief include *denial*, when one aspect of the person's life is not acknowledged; *splitting*, where the person finds it difficult to cope with the positive and negative aspects of a person or a phenomenon, and thus detaches one from the other; and *defence*, where the person's feelings of inadequacy are turned into feelings of empowerment.

Could the psychodynamic approach be used to explain the adoption of conspiracy beliefs as well as delusions? Conspiracy beliefs can be explained by highlighting mechanisms that are amenable to a psychodynamic interpretation. In particular, conspiracy beliefs have been thought to arise from a kind of *projection*, where the enemy identified in the conspiracy theory is a projection of the self. The thesis has first been proposed in the 1960s (Hofstadter, 1964) and has been influential since: it was tested in a study where people's willingness to endorse conspiracy theories was associated with their willingness to engage in conspiracies (Douglas & Sutton, 2011). So, people were asked whether they endorsed a conspiracy theory (such as “The AIDS virus was created in a laboratory”) and whether they would see themselves conspire in the same context (“If you were a scientist, would you have created the AIDS virus?”). These interesting results suggest that at the basis of the acceptance of conspiracy theories there are social cognitive processes that are not pathological. It makes sense that the people who would take part in a conspiracy themselves (“I would do it!”) find it more plausible that there are conspiracies all around (“They did it!”).

Another way in which psychodynamic accounts can shed light into the adoption of conspiracy beliefs concerns the role of motivation. What motivation would there be to adopt a conspiracy belief? One powerful motivation is the *need for uniqueness* which can be described as our desire to be special, different from other people (Lantian et al., 2017). One way for a person to affirm that they are special is to entertain views that are different from, and more interesting and entertaining than, mainstream views: “The conspiracy theory offers the chance of hidden, important, and immediate knowledge, so that the believer can become an expert, possessed of a knowledge not held even by the so-called experts” (Billig, 1987, p. 132). Studies have shown that when people have a greater need for uniqueness, and are more likely to agree with statements such as “I prefer being different from other people”, then they are also more likely to endorse conspiracy theories and believe conspiracy theories more strongly (Lynn & Harris, 1997).

According to psychodynamic theories, the adoption of conspiracy beliefs is not the outcome of cognitive processes that are dysfunctional. Rather, the processes responsible for the adoption of conspiracy beliefs respond to the person's psychological needs, such as the need for self-empowerment and uniqueness, and can be seen as psychologically adaptive in some respect.

## Conspiracy belief as the explanation for an uncertain reality

The explanatory framework of predictive coding has been applied to the adoption and maintenance of clinical delusions and conspiracy beliefs alike. According to predictive-processing theories, conspiracy beliefs can be viewed as *inferences under uncertainty* and, in particular, responses to situations characterised by ambiguity or threat. Belief updating and learning differences are observed in those who adopt a conspiracy theory. This includes a higher sensitivity to changes in the environment and a tendency to predict uncertain events inflexibly. Are those differences a sign of a dysfunction?

The idea behind predictive coding is that the mind is in the business of predicting what comes next: in Hohwy's (2014) words, “the brain is a sophisticated hypothesis-testing mechanism, which is constantly involved in minimizing the error of its predictions about the sensory input it receives from the world” (p. 1). People form expectations about their experiences in line with their general model of the world, a model constructed on the basis of previous experiences. In this process, attention is grabbed by events that are unexpected, because they may signal that the existing model of the world is incomplete or inaccurate. If the model of the world did not predict an important event, then it may need to be upgraded so that it will not fail to predict similar events in the future. When expectations are not met—and thus a prediction error is coded—there is an opportunity to integrate new information in the model of the world, and that can then be used for future predictions.

What happens when prediction-error signalling is disrupted? An event grabs attention but does not deserve it, because the event can actually be predicted by the person's existing model of the world. So, the person is led to make changes to their existing model of the world even if the model is still adequate. This disruption might manifest in the person experiencing an event as particularly salient and thus urgently requiring an explanation, when the event should not be seen as salient and could be explained by the resources already available to the person. This happens because the prediction-error signal is imprecise: it is a warning that something is off, but does not say what is off exactly.

An interesting question is whether the prediction-error theory can tell us also something specific about conspiracy beliefs at a time of crisis. Some work has been done on the increasing levels of paranoia that we observe when a crisis emerges. During the COVID-19 pandemic, it transpired that “people who were more paranoid endorsed conspiracies about mask-wearing and potential vaccines and the QAnon conspiracy theories” (Suthaharan et al., 2021, p. 1190). Can the prediction-error theory make sense of this?

In general, in a crisis people feel threatened and the way they revise their beliefs changes: perhaps unsurprisingly, during the COVID-19 pandemic, the levels of paranoia and anxiety in self-reports soared as a response to the unpredictability of the world. But paranoia increased much more significantly than anxiety, showing that what changed the most was the attitude towards other people. There was increasing concern about other people's intentions. As conspiracy beliefs are based on blaming some powerful individuals or groups for a negatively valued event, the increase in paranoia can help explain why in critical situations conspiracy theories flourish. However, paranoia and conspiracy beliefs do not overlap perfectly and although there are relevant environmental factors affecting both phenomena, paranoia seem to be closely related to personality characteristics such as neuroticism and introversion, whereas conspiracy theories are commonly associated with low trust in authorities (Imhoff & Lamberty, 2018).

The factors that affected belief updating in conspiratorial thinking during the pandemic were environmental: people modified their behaviour in response to the perceived volatility of their environment and also to the measures that authorities put in place to stop the spreading of the virus. This suggests that the observed anomalies in belief updating are unlikely to be evidence of a dysfunction affecting how people process information. Rather, the anomalies seem to be due to an increased tendency in times of crisis to attribute salient events to the (evil) intentions of others. This tendency gives rise to conspiracy beliefs, implausible beliefs that are difficult to shake, and occasionally drive people to make decisions that have harmful consequences for themselves and others, such as the failure to comply with health and safety recommendations or the refusal to be vaccinated against a health threat. Although the consequences of the spreading of conspiracy beliefs during the COVID-19 pandemic as I described them are mostly negative, it is not clear that the beliefs themselves are the output of a dysfunctional mechanism. In some circumstances, whether beliefs are adaptive or maladaptive seems to depend on their contribution to people's chances for survival and reproduction in the environment where they are situated, and the processes responsible for the formation of those beliefs change when the environment changes. It may not always be maladaptive to grow more suspicious of other people's intentions when the world is viewed as threatening and volatile.

## Conspiracy beliefs as an effect of mistrust (and cognitive deficits?)

According to the one-factor theory, the formation and maintenance of conspiracy beliefs can be explained without resorting to a fault in reasoning—epistemic mistrust directed towards sources that are standardly attributed epistemic authority in the environment is sufficient to lead people to endorse conspiracy theories, although what should count as an “epistemic authority” is itself an issue that deserves to be discussed and researched in greater detail than I can do here. According to the two-factor theory, the formation and the maintenance of delusional beliefs cannot be fully explained by epistemic mistrust alone and a fault in reasoning needs to be postulated. The difference between one-factor and two-factor theories means that their supporters will likely offer different answers to the question whether conspiracy beliefs are pathological beliefs in a naturalist sense.

For the one-factor theory, no reasoning deficit needs to be involved in the explanation of the formation or maintenance of conspiracy beliefs. Thus, for one-factor theorists, the answer to the question whether conspiracy beliefs are the output of a cognitive dysfunction is a straightforward one. There is no cognitive dysfunction and thus conspiracy beliefs do not meet the necessary requirement for being pathological beliefs in the naturalist account of disorder. For two-factor theories, the answer to the question whether conspiracy beliefs are the outputs of a cognitive dysfunction is more complicated. That is because two-factor theorists argue that the conspiracy belief is due to the combination of epistemic mistrust and a second factor which amounts to a fault in reasoning—and that can be caused by a cognitive dysfunction.

On one version of the two-factor theory, the second factor is a cognitive deficit, so conspiracy beliefs are the outputs of a cognitive dysfunction. In the deficit version of the two-factor theory, conspiracy beliefs meet the condition for pathological beliefs in a naturalist account of pathological beliefs. In addition to an underlying epistemic mistrust, there is also a deficit that can be described in different ways but is often characterised either as an inability to inhibit implausible hypotheses at the stage of belief adoption, or as an inability to reject a belief that has received disconfirmation at the stage of belief maintenance.

Let's consider the first position, where the deficit is an inability to inhibit an implausible hypothesis. In the two-factor theory, the first factor tells us why people come up with a hypothesis with its distinctive content to account for a significant event and do not accept the “official” explanation of the event. The first factor is often taken to be a form of epistemic mistrust towards the sources of mainstream information. The second factor tells us why the hypothesis, once formulated, is not rejected and is instead adopted as a belief in spite of its implausibility. In the case of conspiracy beliefs emerging at a critical time, the content of the hypothesis is that, even if authorities and other citizens do not acknowledge it, there is a plot by someone powerful and ill-intentioned which explains the crisis (e.g., the Chinese created the coronavirus in a lab in Wuhan). On this view, the hypothesis about the conspiracy is formulated because people do not trust the official account of the event and it is endorsed because people are unable to reject it on the basis of its implausibility.

Coltheart (2007) defends a similar two-factor account of belief formation in the context of clinical delusions. The inability to inhibit implausible hypotheses is a problem with the evaluation of hypotheses leading up to *belief formation*. Due to the tendency to mistrust the official version of the events proposed by the authorities, people actively seek alternative versions of the events. As they lack the capacity to reject hypotheses on the basis of implausibility, they end up endorsing unusual beliefs. If the liberal acceptance of implausible hypotheses is caused by a cognitive dysfunction, then we have our argument for the pathological nature of conspiracy beliefs. However, the problem with relying on this version of the two-factor theory as a justification of the pathological nature of conspiracy beliefs is that the failure to inhibit an implausible hypothesis does not need to be caused by a cognitive deficit, but may also be caused by other factors, including a motivationally biased handling of the evidence.

What can explain the endorsement of an implausible hypothesis? Maybe people are gullible, science-illiterate, or vulnerable to some of the biases I discussed earlier, such as the confirmation bias and the need for uniqueness. Another possibility is that they are sensitive to the need for closure (Webster & Kruglanski, 1994; Roets & Van Hiel, 2007)—which is the need to explain an unsettling event or solve a problem before sufficient evidence can be gathered, due to a low tolerance for uncertainty. In situations where the epistemically authoritative sources cannot provide an explanation or a solution promptly, or consensus is missing, the need for closure may be instrumental to the adoption of conspiracy beliefs. Cognitive biases may lead to accepting a hypothesis even if such a hypothesis is implausible and there is (or there is likely to be in future) robust evidence against it.

Alternatively, one could identify the relevant cognitive deficit with an impairment in belief maintenance, that is, a problem with the assessment of the evidence for and against the conspiracy belief that emerges *after* the endorsement of the implausible hypothesis. A similar position is defended by Coltheart et al. (2010) in the context of delusional beliefs. Suppose that given the unusual nature of the significant events to be explained, it is not problematic to accept an implausible hypothesis as long as it does make good sense of the events. However, when evidence against the conspiracy belief starts accumulating, people should be disposed to suspending judgement, questioning their belief, revising or rejecting the belief altogether, thereby showing responsiveness to external challenges. If this does not happen and the belief perseveres, becoming impermeable to evidence, then a cognitive dysfunction can be appealed to as an explanation of the unshakeability of the conspiracy belief. As with the previous account of the cognitive deficit involved in conspiracy beliefs, the problem with using the two-factor theory as a justification of the pathological nature of conspiracy beliefs is that the failure to revise an unusual belief under the pressure of counterevidence and counterargument does not need to be caused by a cognitive deficit, but can be also due to motivational and reasoning biases that do not amount to a cognitive dysfunction. McKay (2012) offers a persuasive defence of the bias version of this view in the context of delusional beliefs.

To suppose that the adoption or maintenance of the belief is caused by biases is a more economical explanation than to postulate a cognitive dysfunction. It is not uncommon to refrain from giving up an explanation that is important to how people see the world and themselves, especially if giving up the original explanation or endorsing the alternative explanation is significantly costly. After all, accepting an alternative to the conspiracy belief once this has been endorsed requires the person to acknowledge that they have been wrong all along. What the deficit version of the two-factor theory describes as a dysfunction seems to be a common feature that characterises beliefs that are not usually described as either delusional or pathological—such as superstitious and prejudiced beliefs, and everyday instances of self-deception and confabulation (McKay et al., 2005).

## Conspiracy beliefs as an output of biased reasoning

Within the bias version of two-factor models, conspiracy beliefs are explained by a first factor described as epistemic mistrust (Pierre, 2020). As we saw, the mistrust in question is generally shared by well-defined social groups and often directed at institutions—such as the press, the experts, the scientific community, the government, and so on. The second factor is also thought to be predominantly cognitive and is described in terms of reasoning biases which would explain the implausibility and unshakeability of the conspiracy beliefs. If conspiracy beliefs are the outputs of biased reasoning, no dysfunction needs to be posited and conspiracy beliefs are unlikely to be successful candidates for pathological beliefs in a naturalist framework. That is because we would expect the presence of a dysfunction to imply that the cognitive process is damaged to an extent that it cannot satisfactorily fulfil its function, and cannot operate as it should operate, but biases do not need to work that way.

The set of biases constituting factor-two contribute to the adoption of the conspiracy belief and account for its implausibility and unshakeability. Such biases may be accentuated in those who are prone to conspiratorial ideation, but can be observed in everyone, and have different outcomes depending on the context. In some situations it may be beneficial to considering a hypothesis that most people would reject and it may also be the right thing to do to hang onto a belief for which some counterevidence has been found. Two key biases for the adoption of conspiracy beliefs are the intentionality bias and the proportionality bias.

The *intentionality bias* is the tendency to interpret other people's behaviour as deliberate (Rosset, 2008). If an event with bad consequences happens, then it is tempting to believe that someone is to be blamed for bringing that event about and that someone had the intention to do so (“Scientists in a lab in Wuhan engineered coronavirus”). The *proportionality bias* is the tendency to believe that big effects have big causes. If there is a significant event, it must have been caused by something equally significant (“The assassination of John F. Kennedy cannot be the result of a mentally unstable person but must be the outcome of a large-scale conspiracy”). These biases underlie the ways in which people seek to fill the explanatory gap created by the rejection of the official account of the events and build defences for their own theories, contributing to the ways in which processes of belief formation and maintenance operate (see Pierre, 2020 and Douglas et al., 2019).

Although such cognitive tendencies may be accentuated in those who are prone to accepting conspiracy theories, and there are some interesting individual differences, they are strategies for coping with the uncertainty caused by adverse events, and attempts to seek a causal explanation for an existing threat that can help manage the threat. Conspiracy theories often restore a sense of control by imposing meaning to distressing circumstances (Bortolotti et al., 2021).

## Conclusion

On the naturalist account of disorder, what would count as a reason for conspiracy beliefs to count as pathological beliefs is their being caused by a belief formation and maintenance process involving a cognitive dysfunction. But the processes giving rise to the adoption and maintenance of conspiracy beliefs do not need to involve a cognitive dysfunction.

There is one account suggesting a role for a cognitive dysfunction, the deficit version of the two-factor theory, according to which conspiracy beliefs are caused by epistemic mistrust combined with a cognitive deficit. But in the case of conspiracy theories, the bias version of the two-factor theory is more popular than the deficit version—probably because neither reasoning impairments nor psychiatric diagnoses are associated with the adoption or maintenance of conspiracy beliefs as such. We are left with no good reason why the implausibility and unshakeability of a belief should be explained by a cognitive dysfunction rather than by biases—in the context of conspiracy beliefs, as in the context of many other beliefs that have similar surface features.
